# Foraging behavior and age affect maternal transfer of mercury to northern elephant seal pups

**DOI:** 10.1038/s41598-024-54527-6

**Published:** 2024-02-26

**Authors:** Sarah H. Peterson, Michael G. Peterson, Joshua T. Ackerman, Cathy Debier, Chandra Goetsch, Rachel R. Holser, Luis A. Hückstädt, Jennifer C. Johnson, Theresa R. Keates, Birgitte I. McDonald, Elizabeth A. McHuron, Daniel P. Costa

**Affiliations:** 1 Western Ecological Research Center, Dixon Field Station, U.S. Geological Survey, 800 Business Park Drive Suite D, Dixon, CA USA; 2 Institute of Marine Sciences, University of California Santa Cruz, Santa Cruz, CA USA; 3 Department of Ecology and Evolutionary Biology, University of California Santa Cruz, Santa Cruz, CA USA; 4 Louvain Institute of Biomolecular Science and Technology, Université Catholique de Louvain, Louvain-la-Neuve, Belgium; 5 CSS, Inc, Fairfax, VA USA; 6 Centre for Ecology and Conservation, University of Exeter, Penryn, UK; 7 Moss Landing Marine Labs, San Jose State University, Moss Landing, CA USA; 8 Department of Ocean Sciences, University of California Santa Cruz, Santa Cruz, CA USA

**Keywords:** Animal migration, Behavioural ecology, Ecology, Biooceanography, Marine mammals, Environmental impact

## Abstract

Deep ocean foraging northern elephant seals (*Mirounga angustirostris*) consume fish and squid in remote depths of the North Pacific Ocean. Contaminants bioaccumulated from prey are subsequently transferred by adult females to pups during gestation and lactation, linking pups to mercury contamination in mesopelagic food webs (200–1000 m depths). Maternal transfer of mercury to developing seal pups was related to maternal mercury contamination and was strongly correlated with maternal foraging behavior (biotelemetry and isotopes). Mercury concentrations in lanugo (hair grown in utero) were among the highest observed worldwide for young pinnipeds (geometric mean 23.01 μg/g dw, range 8.03–63.09 μg/g dw; *n* = 373); thus, some pups may be at an elevated risk of sub-lethal adverse health effects. Fetal mercury exposure was affected by maternal foraging geographic location and depth; mercury concentrations were highest in pups of the deepest diving, pelagic females. Moreover, pup lanugo mercury concentrations were strongly repeatable among successive pups of individual females, demonstrating relative consistency in pup mercury exposure based on maternal foraging strategies. Northern elephant seals are biosentinels of a remote deep-sea ecosystem. Our results suggest that mercury within North Pacific mesopelagic food webs may also pose an elevated risk to other mesopelagic-foraging predators and their offspring.

## Introduction

The maternal transfer of environmental contaminants to offspring is the initial delivery of contamination to younger age classes, making them vulnerable to adverse health effects because this transfer occurs during critical development periods. Substantial research has been conducted on environmental contaminant exposure, accumulation, and, in some cases, effects on wild animal populations^1–5^. Despite knowledge of the potential risk posed by contaminant exposure to young animals, the factors influencing the extent to which the contaminant burden of a breeding mammalian female is passed to her offspring, and the risk from toxicological effects as a result of that transfer, have been understudied. For birds, reptiles, amphibians, and bony fishes, a portion of the maternal burden is transferred into one or more eggs, with the degree of maternal contamination affecting the transfer rate of contaminants to offspring^6–9^. For example, the proportion of mercury transferred into bird and frog eggs decreases with increasing maternal mercury contamination^6,7,9^, whereas fish transfer a higher proportion of their mercury burden to their offspring as maternal mercury concentrations increase^8^. Transfer of contaminants from mammalian females to offspring, however, occurs both during gestation via blood from the placenta and during lactation via milk from the mammary gland^10–14^, creating challenges for clarifying the different maternal transfer mechanisms and evaluating linkages between offspring and maternal mercury exposure.

Differences in contaminant concentrations among reproductive females may result in variation in the contaminant load transferred to offspring and their subsequent risk of adverse health effects. Individual variation in contaminant concentrations can result from different foraging behaviors that are associated with regional and temporal differences in contaminant exposure from prey^1,15–20^. Furthermore, maternal age^16,21^ and whether or not females have previously produced offspring^22,23^ may also contribute to variability in individual maternal contaminant concentrations. Contaminant concentrations in adult animals can also vary markedly with changes in body condition^24–26^, which has particular importance in some marine mammal species where adult females undergo periods of fasting during gestation and lactation^24,27–29^. For lipophilic persistent organic pollutants, primiparity can influence the magnitude of the contaminant concentrations passed to offspring, with the highest contaminant transfer to the first offspring produced^22,23^. However, knowledge about how primiparity influences the transfer of heavy metals such as mercury to offspring is limited.

Mercury bioaccumulation through diet is prevalent worldwide in freshwater and marine ecosystems and maternal transfer of mercury to offspring occurs across vertebrate taxa from bony fishes to mammals^7,8,10,11,30,31^. Mercury bioaccumulation can have neurotoxic and immunotoxic effects^3–5,32–35^. Mesopelagic species may be particularly vulnerable to mercury bioaccumulation because mercury is pervasive in this zone within marine food webs. In association with the oxygen minimum zone, inorganic mercury (Hg^II^) within the mesopelagic can be converted to methylmercury by anaerobic bacteria and become bioavailable for incorporation into deep ocean food webs^36,37^. Consequently, the mesopelagic zone (200–1000 m) contains higher concentrations of both total mercury and methylmercury, which is the bioaccumulative form of mercury that presents the greatest risk for adverse health effects, than either the epipelagic (0–200 m) or the zones deeper than 1000 m^15,38–40^. Moreover, even if current inorganic mercury emissions, such as those from coal-fired power plants, were to immediately cease, mercury concentrations in marine food webs are expected to continue increasing due to the substantial time lag (decades to centuries) to reach equilibration between mercury levels in the atmosphere and the oceans^41–44^. These factors heighten the future risk of mercury exposure to mesopelagic-foraging marine predators and their offspring.

Northern elephant seals (*Mirounga angustirostris*) provide a unique opportunity to study the factors affecting the transfer of mercury from mother to offspring in mesopelagic predators. Northern elephant seals forage almost exclusively within the mesopelagic zone during their biannual foraging trips across the North Pacific Ocean, with individual adult females demonstrating substantial variability in diving behavior (i.e*.*, median foraging dive depth during the day can differ among individuals by nearly 300 m) and geographic location^16,45,46^. Although some individual northern elephant seals show dietary specialization, northern elephant seals are considered a generalist predator of mostly mesopelagic fishes and squids^47,48^. Annually, adult female northern elephant seals undergo an approximately 7-month-long foraging trip, upwards of 10,000 km in total distance traveled, during which gestation of the pup occurs. Females then return to land to give birth to a single pup and nurse their pup for 3 to 4 weeks, which they do entirely while fasting. Because the maternal transfer of mercury to offspring for marine mammals is thought to primarily occur during gestation and to a lesser extent through lactation^12–14,49^, the foraging behavior of adult female elephant seals is likely to have the most direct impact on the risk of mercury exposure and bioaccumulation for young and developing pups. If maternal transfer of mercury to offspring is reflective of maternal bioaccumulation^50^, then the offspring of some individuals may be more at risk for adverse effects from mercury exposure as a consequence of the increased mercury contamination of some females because of their foraging strategy^16^.

We used free-ranging northern elephant seals as a model species to investigate maternal transfer of mercury to pups in relation to maternal traits, including maternal blood mercury concentrations, and to quantify the links between pup lanugo mercury concentrations and maternal mesopelagic foraging behavior during gestation. We satellite tracked known-age adult females at sea during their 7-month-long foraging trip to determine maternal foraging strategies (geographic locations and diving depths) and quantified mercury concentrations in maternal blood and their pups’ blood and lanugo (hair grown in utero) early in lactation when seals were hauled out. We then tested how the maternal transfer of mercury to pups is influenced by additional maternal traits, such as maternal age, as well as for intra-year variability by including additional lanugo samples from more than 300 pups of known adult females that we did not handle or track at sea.

## Materials and methods

### Experimental design

#### Field sampling

To quantify the relationships between pup mercury (Hg) concentrations and both maternal Hg concentrations and maternal at-sea behavior, we sampled mother and pup pairs during the 2013 to 2022 breeding seasons at the Año Nuevo colony (Año Nuevo Reserve, San Mateo County, California, USA). All animal handling protocols were approved by the Institutional Animal Care and Use Committee (IACUC) at the University of California, Santa Cruz, and were conducted under National Oceanic and Atmospheric Administration (NOAA) Marine Fisheries Service permits 14636, 19108, and 23188. All procedures involving animals were conducted in accordance with the relevant guidelines and regulations of the IACUC protocol and the NOAA permits, and methods are reported in accordance with ARRIVE guidelines 2.0 (https://arriveguidelines.org/arrive-guidelines). To record spatial and diving behavioral metrics, we deployed satellite transmitters (e.g., Wildlife Computers, Bellevue, Washington, USA: SPOT5; Sea Mammal Research Unit, St. Andrews, Scotland: SRDL-CTD) and time-depth recorders (e.g., Wildlife Computers MK9) on adult female seals at the end of the molting period just prior to females returning to sea for their long foraging trip. When females returned to land for breeding (*n* = 82 foraging trips from 71 unique adult females), we targeted day 5 post-parturition (73% sampled on day 5; range 4–9 days post-parturition) to recover instruments and collect tissue samples from females (whole blood) and their pups (whole blood and lanugo). These females were on the beach various lengths of time before parturition; adult females were sampled on average 9 ± 2 days (range: 5–15 days) after returning from their foraging trip. In order to test whether blood Hg concentrations and blood Hg burdens change in pups during the lactation period, we collected paired whole blood samples from a subset of 9 pups during both early lactation (5 days post-parturition) and late lactation (23 days post-parturition) to quantify changes in blood Hg concentrations and estimate the circulating pup blood Hg burden at those time periods. In a third subset of pups, we collected lanugo (but not blood) from pups produced by flipper-tagged adult females that were at the end of nursing or recently weaned but not yet fully molted.

Adult females were chemically immobilized using previously described procedures^45,46^ to deploy or recover instruments, and collect morphometric measurements (i.e., body mass, standard length). Whole blood, hereafter blood, and lanugo samples were collected and processed following standard and previously published protocols for Hg analysis^16,51^. In brief, blood samples were collected from the extradural vein into vacutainers containing sodium heparin and placed on wet ice in the field. Upon return to the laboratory, blood was gently re-homogenized, an aliquot was transferred into a polypropylene cryovial, and samples were frozen at −20 °C until Hg analysis. A separate aliquot of whole blood was centrifuged, and an aliquot of red blood cells was placed into a polypropylene cryovial until preparation for stable carbon and nitrogen isotope analysis, following established protocols^16,52^. Pup lanugo samples were collected from the dorsal midline using cordless clippers and stored in small paper envelopes or whirl–pak® bags. Lanugo samples were stored in a dark cupboard until they were washed and dried prior to Hg determination.

#### Hg determination

Tissue samples were analyzed for Hg at the U.S. Geological Survey Dixon Field Station Mercury Lab using either a Milestone DMA-80 Direct Mercury Analyzer (Milestone, Shelton, Connecticut, USA) or a Nippon Instruments MA-3000 Direct Mercury Analyzer (Nippon Instruments Corporation, Tokyo, Japan) using U.S. Environmental Protection Agency Method 7473^53^. Thawed blood samples were carefully inverted multiple times to rehomogenize before an aliquot was removed for analysis. Blood (~ 100 µL) and lanugo (4–8 mg) samples were weighed to the nearest 0.01 mg and then placed in the Hg analyzer. Total mercury (THg) concentrations were generated using wet weight (μg/g ww) for blood samples and dry weight (μg/g dw) for lanugo samples. Recoveries (arithmetic mean ± SE) were 100.2 ± 0.4% (*n* = 85) for certified reference materials (DORM-3, DORM-4, TORT-3, DOLT-3, and DOLT-4) and 100.5 ± 0.3% (*n* = 69) for continuing calibration verifications. The relative percent difference for duplicate samples averaged 3.8 ± 0.4% (*n* = 34) for lanugo and 2.0 ± 0.5% (*n* = 23) for blood.

#### Stable carbon and nitrogen isotope determination

We quantified *δ*^13^C and *δ*^15^N isotope values in maternal red blood cells, following established methods^16,52,54^. Briefly, lyophilized red blood cells (~ 0.5 mg) were analyzed using an elemental analyzer (Carlo Erba NE2500) coupled to an isotope ratio mass spectrometer (ThermoFinnigan DELTAplus XP) at the University of California, Santa Cruz light stable isotope laboratory. Average experimental precision, taken as the mean of all within-run standard deviations for in-house standards, was 0.10‰ for *δ*^13^C and 0.08‰ for *δ*^15^N.

### Statistical analyses

We used pup THg concentrations to test the influence of maternal age, year, and at-sea foraging behavior on maternal transfer, and to examine adult female repeatability in maternal transfer to offspring. Specifically, we (a) quantified the relationship between pup and maternal THg concentrations, (b) determined how pup blood THg concentrations and the total quantity of THg in pup blood (blood THg burden) changed across the nursing period, (c) examined if the THg concentration in pups of primiparous females (first offspring) was higher than pups born to the same female in subsequent years, (d) quantified how repeatable pup THg concentrations were from the same female over time, (e) examined how maternal transfer of THg was related to maternal age and year, and (f) described the relationship between pup lanugo THg concentrations and maternal foraging behavior (Table 1). All analyses were conducted in the statistical program R (version 4.0.5)^55^. Model performance and the assumption of normality was assessed by checking the distribution of residuals. Total Hg concentrations were log_e_-transformed for all analyses, unless otherwise specified, to improve normality of residuals. Statistical significance was set at *p* ≤ 0.05.

**Table 1:** Sample sizes by analysis with the unique characteristics of each dataset.

Analyses	*n*	Y-axis	X-axis	Dataset comments
a) Tissue [THg] correlations	80	Pup lanugo [THg]	Maternal blood [THg]	Paired pup lanugo and maternal blood
	65	Pup lanugo [THg]	Maternal blood [THg]	Paired pup lanugo, pup blood, and maternal blood
	65	Pup blood [THg]	Maternal blood [THg]	Paired pup lanugo, pup blood, and maternal blood
	66	Pup lanugo [THg]	Pup blood [THg]	
b) Changes in pup THg over lactation	9	∆ pup blood [THg]	∆ body mass	
	9	∆ pup blood THg burden	∆ body mass	
c) Pup [THg] from primiparous females vs. subsequent pups	21	Pup lanugo [THg] (subsequent pup)	Pup lanugo [THg] (first pup)	Subsequent pups sampled 1 to 4 years after the first pup
d) Repeatability of [THg] in successive pups of known females	90	Repeatability value for pup lanugo [THg]	–	220 total pups sampled; 2–4 pups per known female
e) Effects of maternal age and year	334	Pup lanugo [THg]	Maternal age; year	Pups of 221 unique known-age females
f) Effects of maternal foraging behavior	53	Pup lanugo [THg]	Maternal at-sea foraging variables; age	Includes maternal stable isotopes, at-sea tracking and diving data

Total mercury concentrations [THg] were quantified in lanugo samples from northern elephant seal (*Mirounga angustirostris*) pups and whole blood (hereafter, blood) samples from pups and paired adult females.

Changes (∆) in pup blood [THg], pup blood THg burden, and body mass were calculated by taking the final value/initial value.

#### Pup and maternal tissue THg correlations

Blood THg concentrations in adult females and pups change over lactation^12,13,26^, whereas lanugo THg concentrations solely represent gestational transfer of THg. Using a subset of samples where we had paired THg concentrations in pup lanugo, pup blood, and maternal blood from early in lactation, we conducted linear regression analyses to assess maternal THg transfer and determine if both pup whole blood THg concentrations as well as pup lanugo THg concentrations could be estimated from maternal blood THg concentrations (*n* = 65). We also analyzed lanugo THg concentrations relative to maternal blood THg concentrations for a larger paired dataset of mothers and pups (*n* = 80) from early in lactation. Within pups, we examined if lanugo THg concentrations could predict blood THg concentrations early in lactation. Because it is not appropriate to simply invert an equation generated from linear regression^56^, we reversed the axes and reran analyses to generate additional equations. For example, our intent was to provide equations that could be used in the future to estimate maternal blood THg concentrations from pup lanugo THg concentrations as well as to estimate pup lanugo THg concentrations from maternal blood THg concentrations.

#### Change in pup blood THg concentrations and blood THg burdens during lactation

Although blood THg concentrations may decline as young animals rapidly increase in body mass^12,57^, the quantity of THg (burden) in pup blood may increase as pups acquire THg through milk via lactation^12^. To compare how pup THg blood concentrations and pup blood THg burdens changed from early to late in lactation, we calculated the change in blood THg concentrations as well as the change in estimated blood THg burden for a subset of 9 pups sampled at day 5 post-parturition and day 23 post-parturition. We determined the blood THg burden (μg Hg) based on individual blood THg concentrations (μg/g), estimates of blood volume (mL), and the specific gravity of blood. Blood volume was estimated as 100 ml/kg pup body mass^58^. The specific gravity of blood was based on individual pup hematocrit measurements (hematocrit was quantified as the proportion of red blood cells within whole blood using standard microhematocrit centrifugation techniques) and estimated to be: 0.089 × hematocrit + 1.018 for elephant seal pups^59^. From this, we generated the following equation to estimate blood THg burdens early and late in lactation for growing pups: μg Hg = (pup blood THg μg/g ww) × (pup mass in kg × 100 ml blood/kg pup) × (0.089 × hematocrit + 1.018).

For both THg concentrations and blood THg burdens, we then conducted paired *t*-tests to examine differences between early lactation and late lactation. Additionally, we tested whether the change in blood THg concentration or blood burden (final/initial) was related to the change in pup body mass over the 18 days (final mass/initial mass) using linear regression.

#### Pup THg concentrations from primiparous females versus subsequent pups

We tested if pups of primiparous females had higher lanugo THg concentrations than subsequent pups of those same females. Using an extensive resighting database, primiparous females were defined as those observed for the first time during a breeding season with a pup; females were not considered primiparous if they had been observed during a prior breeding season, irrespective of whether they were seen with a pup at that time. The modal age of primiparity in northern elephant seals is 4 (56.6% of primiparous females), with almost all seals having their first pup between 3–6 years of age^60,61^. For 14 females, we sampled lanugo from the presumed first pup and the pup from the subsequent year. For 7 additional females, we sampled the presumed first pup and a pup born 2 to 4 years after the first observed pup. We ran a paired one-tailed *t*-test with all 21 females to test the hypothesis that pups of primiparous females would have higher lanugo THg concentrations (untransformed THg concentrations). We ran an additional one-tailed *t*-test with only the 14 seals from consecutive years and confirmed that excluding non-consecutively collected samples did not influence the results (*t* = 1.78, *df* = 13, *p* = 0.95).

#### Repeatability of THg concentrations in the pups of adult females

For an individual adult female, pup lanugo concentrations were considered repeatable when the lanugo THg concentrations were demonstrably more consistent through time among her pups compared to the sampled population. We quantified the repeatability of pup lanugo THg concentrations at the population level using a subset of all marked females with ≥ 2 pups sampled between the 2013 and 2022 breeding seasons (*n* = 90 adult females; 2–4 pups per female). We estimated the confidence interval around the population level repeatability estimate from 1000 bootstraps with the *rptR* package^62,63^. Likelihood ratio tests were used to test for the importance of the random effect (maternal identification). Repeatability (R) ranges from 0 to 1, with values closer to 1 indicating greater repeatability; values > 0.75 were considered strongly repeatable, and values of 0.5–0.75 were considered moderately repeatable^64,65^. Additionally, we estimated the individual adult female level repeatability of pup lanugo THg concentrations based on the between-individual variability and the residual variance for each individual, following a previous study^64^. After we obtained individually calculated repeatability values, we conducted Spearman’s rank correlation analyses to assess if the individual repeatability values were related to the number of pups we sampled per female or the maximum number of years between the first and last samples from a female.

#### Influence of maternal age and year on pup lanugo THg concentrations

To determine if elephant seal fetuses experienced variability in Hg exposure as a function of maternal age or year, we quantified THg concentrations in pup lanugo of 334 nursing or recently weaned elephant seal pups from 221 known-age adult females between 2013 and 2022. To describe the relationship between pup lanugo THg concentrations and maternal age, while accounting for year, we fit a generalized additive mixed model (GAMM) using the *mgcv* R package^66^, which does not restrict the model to a specific polynomial function (e.g., linear, quadratic, cubic). The interpretation of results and model selection for GAMMs is similar to generalized linear models^66–68^, with linear predictors that are an additive combination of local smoothers. We included a global smoother (thin plate regression spline) for maternal age and a random effect for individual female to account for repeated pup samples from the same female. We included individual smoothers for each of the 9-factor levels for year to account for and describe potential differences among THg concentrations in pup lanugo among years, after accounting for maternal age and differences among individual females. We ran models using default parameters for the link function (‘identity’) and the smoothing basis dimension (*k)*, with model coefficients and smoothing parameters estimated using restricted maximum likelihood (REML). We then extracted model-estimated predictions for mean and 95% CI pup lanugo THg concentrations by age for the breeding season 2021, where we had the most data (25% of samples). Secondarily, we predicted mean and 95% CI pup lanugo concentrations for each breeding season for 6-year-old females, the most common maternal age in the dataset (17% of samples), to illustrate differences among years.

#### Pup lanugo THg concentrations related to maternal foraging behavior

To test whether pup lanugo THg concentrations (i.e., Hg exposure in utero) could be directly linked to maternal at-sea behavior, we used a subset of adult females with complete tracking and diving records and corresponding pup lanugo THg concentrations sampled during breeding seasons from 2013 to 2021 (*n* = 53 pups from 48 adult females). Our analysis generally followed a previously published method^16^ with both a clustering analysis to test for differences in pup lanugo THg concentrations at a broad scale and an information criterion approach to examine individual predictor variables to explain pup lanugo THg concentrations at the level of individual pups.

From the time-depth recorder records, we summarized the median and the 90th percentile depths of day and night putative foraging dives for each seal^16^. We smoothed satellite tracks and linearly interpolated satellite tracking data to obtain a location every 8 h using either the *crawl* R package^69,70^ or the *foieGras* R package^71,72^. Additionally, we calculated the median distance between all locations and the continental shelf, as defined by the 200 m isobath within the ETOPO 1 Arc-Minute Global Relief Model^73^, the farthest north location reached during the foraging trip (northernmost latitude), and the farthest west location reached during the foraging trip (westernmost longitude). Any locations interpolated at the end of the foraging trip that fell on land before the transmitter was recovered were presumed to have occurred on the continental shelf.

We used a Principal Components Analysis (PCA) to reduce a set of 10 tracking and diving variables and stable isotopes (Table 2) into unrotated factors (retaining the top 4 principal components that comprised 83% of the variance) that could then be clustered into similar groups based on intra-cluster inertia (allowing 2–10 possible clusters), using the *HCPC* function (hierarchical clustering on principal components) within the *FactoMineR* package^74,75^. Once each pup lanugo sample was assigned to a maternal foraging behavior cluster, we tested for differences in pup lanugo THg concentrations (untransformed THg concentrations) among clusters using linear mixed models (*lme4* R package^76,77^) with cluster as a fixed effect and adult female identification as a random effect to account for some repeatedly sampled females. Using the *car* R package, we quantified significance using type II *F*-tests with Kenward-Roger approximation for degrees of freedom^78^. We examined differences between behavioral clusters using post-hoc pairwise tests on model-generated least squares mean pup lanugo THg concentrations.

**Table 2:** Variables used in foraging behavior cluster analysis.

Variable	Northerly/more coastal	Pelagic/shallower diving	Pelagic/deeper diving	Overall
Pup lanugo THg (µg/g dw)	21.66 ± 7.97^a^ (*n* = 17)	26.01 ± 5.24^ab^ (*n* = 23)	29.45 ± 8.05^b^ (*n* = 13)	25.5 ± 7.4 (*n* = 53)
Northernmost latitude (º)	53.5 ± 4.4*	47.3 ± 3.1*	46.7 ± 1.3*	49.2 ± 4.4
Westernmost longitude (º)	211.9 ± 12.9*	195.7 ± 10.7	190.6 ± 7.0*	199.7 ± 13.7
Median distance to continental shelf (km)	341.9 ± 214.8*	998.5 ± 188.0*	963.5 ± 135.4*	779.3 ± 354.5
Median day foraging dive depth (m)	574 ± 50*	635 ± 32	686 ± 36*	628 ± 58
90th quantile of day foraging dive depths (m)	688 ± 31*	786 ± 69	901 ± 54*	783 ± 97
Median night foraging dive depth (m)	466 ± 35	471 ± 52	496 ± 36	476 ± 44
90^th^ quantile of night foraging dive depths (m)	626 ± 55	625 ± 51*	723 ± 80*	649 ± 73
Maternal *δ*^13^C (red blood cells; ‰)	− 19.4 ± 0.4	− 19.5 ± 0.2	− 19.5 ± 0.2	− 19.5 ± 0.3
Maternal *δ*^15^N (red blood cells; ‰)	15.0 ± 0.9*	14.6 ± 0.6	14.4 ± 0.2	14.7 ± 0.7

At-sea geographic locations and diving depths for adult female northern elephant seals (*Mirounga angustirostris*; *n* = 53 pups from 48 unique females) from the Año Nuevo Colony (California, USA) were used to identify 3 clusters of foraging behaviors. Total Hg concentrations (THg µg/g dry weight) in lanugo of pups born to satellite-tracked adult female northern elephant seals (sampled from 2013 to 2021) were compared among foraging clusters (model-generated least squares mean ± SE).

Different letters for pup lanugo THg concentrations indicate significant differences (*p* < 0.05) among clusters. All variables used in the cluster analysis were summarized for each cluster as well as the overall dataset and are presented as arithmetic mean ± SD.

Asterisks (*) indicate the predictor variables that were significant in distinguishing each cluster (different from the overall mean value for each variable).

To describe maternal foraging behavior at the level of the individual pup and determine which individual predictor variables were best able to explain variability in pup lanugo THg concentrations (untransformed THg concentrations), we first started with all variables from the cluster analysis described above as well as maternal age and year. We checked all variable pairs and because of strong correlations with other variables (Pearson’s product moment correlation, *R* > 0.70), we did not include the median distance to the continental shelf, *δ*^15^N, and the 90th quantile of the day foraging dive depths. At this point, we created a set of candidate general linear models including all combinations (*n* = 256 models) of the following 8 variables. We included a categorical variable for the year of the foraging trip and continuous numerical variables for northernmost latitude (º), westernmost longitude (º), *δ*^13^C (‰), the median depth of day foraging dives (m), the median depth of night foraging dives (m), the 90th quantile of night foraging dive depths (m), and maternal age. We had 8 females for which we did not have a confirmed birth year, 6 of which were handled for the first time and flipper tagged as adults, and 2 that were handled initially and flipper tagged as juveniles. For these 8 females, we assigned 5 as the age for the year a female was first identified as an adult on the colony, 2 as the age for the year a female was identified as a juvenile, and then estimated the age for the female at the end of the foraging trip when her pup was sampled using the initial sighting.

We ranked candidate models based on Akaike information criterion values corrected for small sample sizes (AIC_c_). We considered the biological importance of all models within a ΔAIC_c_ ≤ 2.0 of the top (highest-ranked) model^79,80^. For each variable in the top model, we calculated evidence ratios by dividing the Akaike weight of the top model by the Akaike weight of the same model without the variable of interest. To account for model uncertainty, we generated model-averaged predictions for pups of 6-year-old females (34% of samples) during the 2013 breeding season (23% of samples), holding all other non-focal continuous variables at their median value.

### Ethics declarations

The authors have complied with ARRIVE guidelines.

## Results

Pup lanugo THg concentrations from simultaneously sampled mother and pup pairs ranged from 10.07–44.26 μg/g dw (geometric mean: 26.38 μg/g dw; *n* = 81) and pup blood THg concentrations ranged from 0.09–0.35 μg/g ww (0.19 μg/g ww; *n* = 67). Maternal blood THg concentrations ranged from 0.18–0.95 μg/g ww (0.37 μg/g ww; *n* = 81). Pup lanugo THg concentrations from all sampled elephant seal pups, including pups that were sampled early in lactation, late in lactation, or were recently weaned, ranged from 8.03–63.09 μg/g dw (23.01 μg/g dw; *n* = 373)^81^.

### Pup and maternal tissue THg correlations

**Figure 1:**
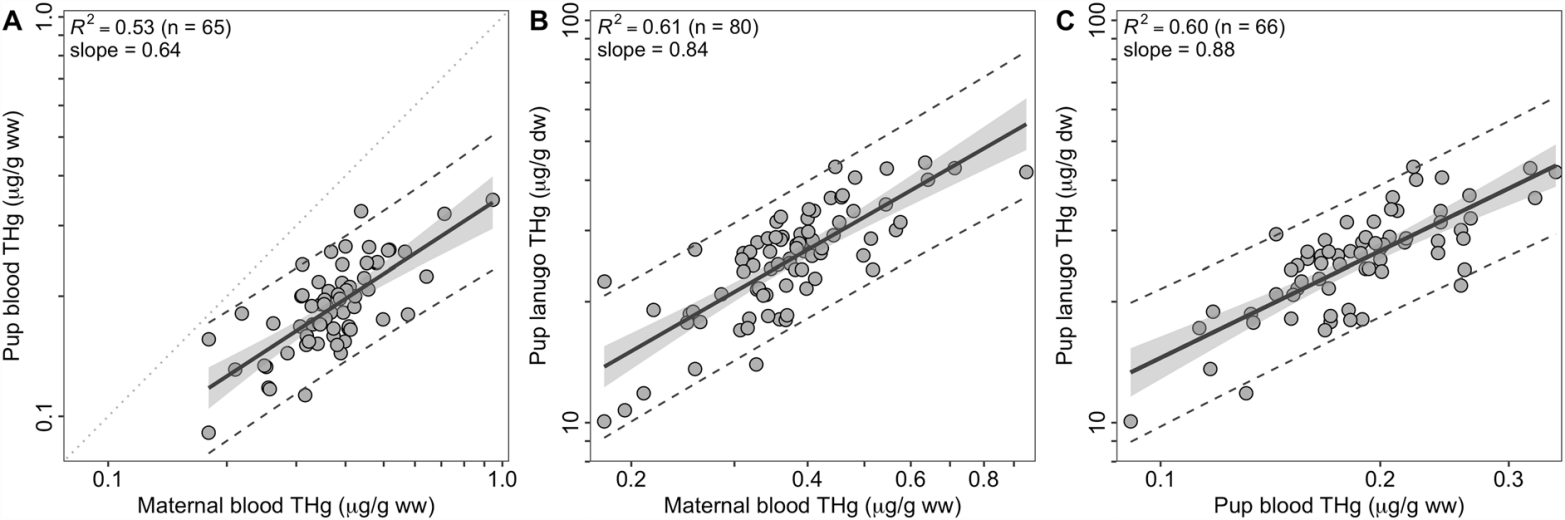
Pup and maternal tissue mercury relationships. Total mercury (THg) concentrations in the lanugo (hair grown in utero) of pups and whole blood of adult female northern elephant seals (*Mirounga angustirostris*) and their pups that were sampled, on average, 5 days after pups were born. (**A**) Pup whole blood THg concentrations (µg/g ww) were correlated with maternal whole blood THg concentrations (µg/g ww). The gray stippled line is the 1:1 line. (**B**) Pup lanugo THg concentrations (µg/g dw) were correlated with maternal whole blood THg concentrations (µg/g ww). (**C**) Pup lanugo concentrations (µg/g dw) were correlated with pup whole blood concentrations (µg/g ww). All three panels show slopes of < 1. Shading indicates standard error around the slope of the regression line, and dashed lines indicate the 95% prediction interval. Statistical analyses were conducted on log_e_-transformed THg concentrations but the relationships are plotted on log_10_ axes for visualization. Note that the x-axis differs for blood THg concentrations of pups and adult females. Refer to the Results for the equations.

Pup lanugo THg concentrations were positively correlated with maternal blood THg concentrations (*R*^2^ = 0.61, *p* < 0.001, slope = 0.64, *n* = 80 pairs; Fig. 1A). We estimated a 300% increase in pup lanugo THg concentrations between the minimum and the maximum observed maternal blood THg concentrations (0.18–0.95 μg/g ww). However, the slope of the relationship was < 1 on a natural log scale, indicating that the proportion of maternal THg that was transferred to pups decreased as maternal blood THg concentrations increased. For example, an increase of 50% in maternal blood THg concentrations resulted in a 40% increase in lanugo THg concentrations, based on the following equation: log_e_(pup lanugo [THg µg/g dw]) = (log_e_(maternal blood [THg µg/g ww]) × 0.83620) + 4.05771. After reversing the axes to estimate maternal blood THg concentrations from pup lanugo (*R*^*2*^ = 0.61, *p* < 0.001), we obtained the following equation: log_e_(maternal blood [THg µg/g ww] = log_e_(pup lanugo [THg µg/g dw]) × 0.72791) − 3.34161.

For the subset of data where we had paired pup lanugo, pup blood, and maternal blood (*n* = 65), we observed a similar relationship early in lactation between pup lanugo THg concentrations and maternal blood THg concentrations (*R*^2^ = 0.56, *p* < 0.001, slope = 0.78) than between pup blood THg concentrations and maternal blood THg concentrations (*R*^2^ = 0.53, *p* < 0.001, slope = 0.64; Fig. 1B).

We observed a moderate positive relationship between pup lanugo THg and pup blood THg concentrations at approximately 5 days post-parturition (*R*^2^ = 0.60, *p* < 0.001, *n* = 66, slope = 0.88; Fig. 1C). The slope of the relationship was < 1, indicating that pups with lower blood THg concentrations had sequestered proportionally more Hg into their lanugo than pups with higher blood THg concentrations. An increase of 240% in lanugo THg concentrations resulted from a 300% increase in pup blood THg concentrations (from 0.10 to 0.40 μg/g ww), based on the following predictive equation: log_e_(pup lanugo [THg µg/g dw]) = (log_e_(pup blood [THg µg/g ww]) × 0.88140) + 4.70330. We then reversed the axes and reran the analysis to obtain the equation to estimate pup blood THg concentrations at day 5 post-parturition from pup lanugo THg concentrations, because pup lanugo samples are more accessible for sampling and lanugo does not grow after birth. An increase of 150% in pup blood THg concentrations (0.10 to 0.25 μg/g ww) resulted from a 300% increase in pup lanugo THg concentrations (10 to 40 μg/g dw), based on the following equation: log_e_(pup blood [THg µg/g ww]) = (log_e_(pup lanugo [THg µg/g dw]) × 0.67742) −3.86091.

### Change in pup blood THg concentrations and blood THg burdens during lactation

**Figure 2:**
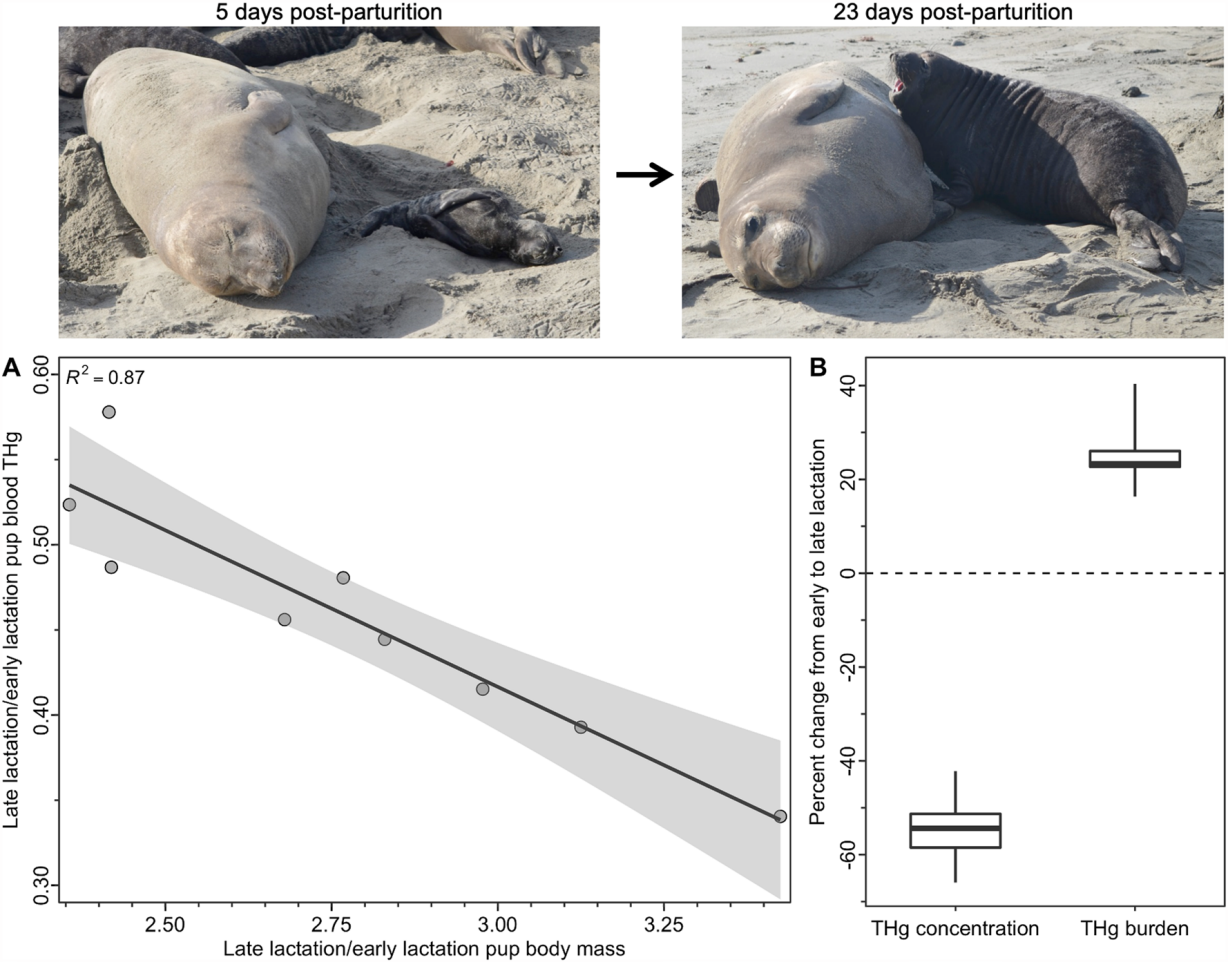
Change in pup blood mercury concentrations and burdens during lactation. (**A**) Northern elephant seal (*Mirounga angustirostris*) pups increased in mass by 136% to 243% (mean 178%) from 5 days post-parturition (early lactation) to 23 days post-parturition (late lactation) with a corresponding decrease in circulating blood total Hg (THg) concentrations. Shading indicates standard error around the slope of the regression line. (**B**) As pup body mass increased from early to late lactation, circulating pup blood THg concentrations decreased 54%. Nevertheless, the absolute quantity of THg in pup blood (blood THg burden) during this same time period increased by 25% (refer to Materials and Methods for these calculations). In each boxplot, the box delineates the interquartile range, the horizontal line shows the median value, and the whiskers encompass the full range of data. Photo credits: S.H. Peterson.

Individual pup blood THg concentrations (*n* = 9) decreased by 54.3 ± 7.1% (arithmetic mean ± SD; range: 42.2–66.0%) over the 18 days from early lactation (0.20 ± 0.05 μg/g ww; range 0.15–0.33 μg/g ww) to late lactation (0.09 ± 0.01 μg/g ww; range 0.07–0.11 μg/g ww; *t* = 7.85, *p* < 0.001). In contrast, we estimated that the quantity of THg in the pup blood over the same 18-day period increased by 25.1 ± 7.5% (16.3–40.3%; *t* = 10.76, *p* < 0.001), as pups grew from a mean 45.3 ± 4.9 kg to a mean 125.0 ± 13.1 kg (Fig. 2). At day 5 post-parturition, we estimated a range of 711.3–1392.5 μg THg (939.1 ± 191.9 μg THg) in pup blood, and at day 23 post-parturition, we estimated a range of 877.3–1619.8 μg THg (1170.1 ± 212.7 μg THg) in pup blood. The proportion by which the pup blood THg concentration decreased over the 18 days was strongly related to the proportional increase in pup body mass (*R*^2^ = 0.87, *p* < 0.001; Fig. 2), although the proportional increase in the blood THg burden was unrelated to the proportional increase in pup body mass (*R*^2^ = 0.16, *p* = 0.28).

### Pup THg concentrations of primiparous females versus subsequent pups

**Figure 3:**
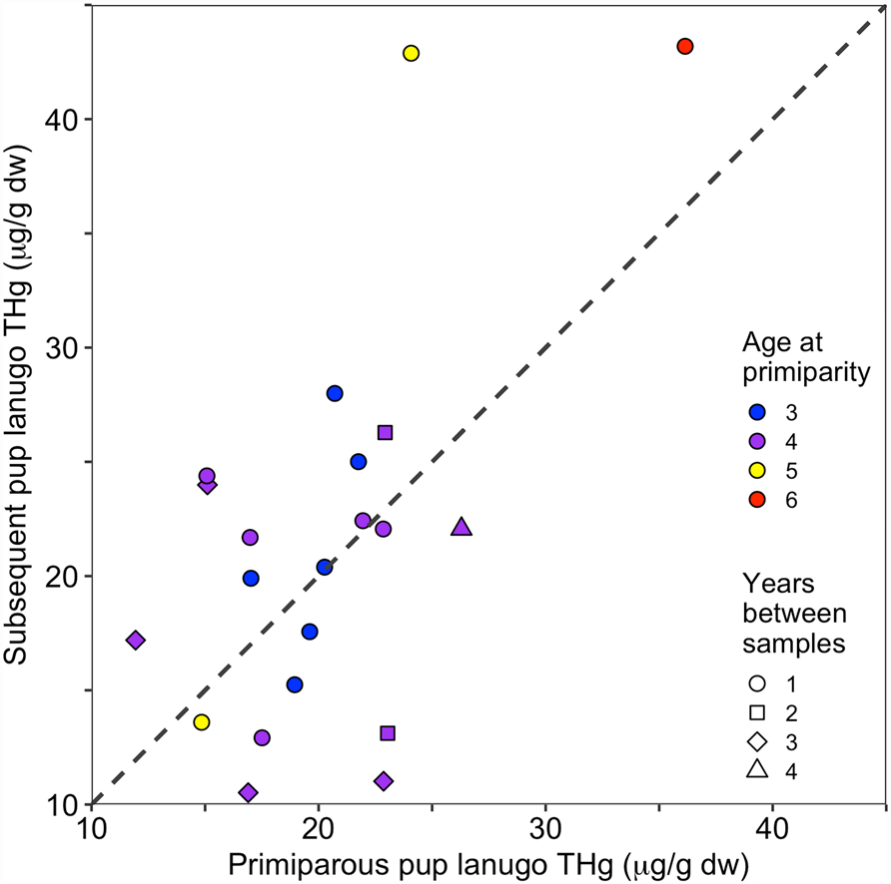
Primiparity and maternal transfer of mercury. The total Hg (THg) concentrations in pup lanugo (hair grown in utero) from primiparous (first time giving birth) female northern elephant seals (*Mirounga angustirostris*; *n* = 21) were not consistently higher or lower than the lanugo THg concentrations from pups of those same adult females born in subsequent years (1 to 4 years later). Colors indicate the age of the female at primiparity (the age when a female had her first pup; 3 to 6 years of age) and the symbol indicates how many years later the subsequent pup was sampled after the primiparous pup was sampled (1 to 4 years later).

Primiparous females were between 3 and 6 years old when observed with their first pup (mean ± SD: 3.9 ± 0.8 years old). The pups of primiparous females did not consistently have higher lanugo THg concentrations than pups of the same females produced 1 to 4 years later (*t* = 0.82, df = 20, *p* = 0.79; Fig. 3). In fact, 57% of females had a lower THg concentration in their first pup than a subsequently born pup and 74% of females sampled in consecutive years (first and second born pups) had a lower THg concentration in the first observed pup.

### Repeatability of THg concentrations in the pups of adult females

**Figure 4:**
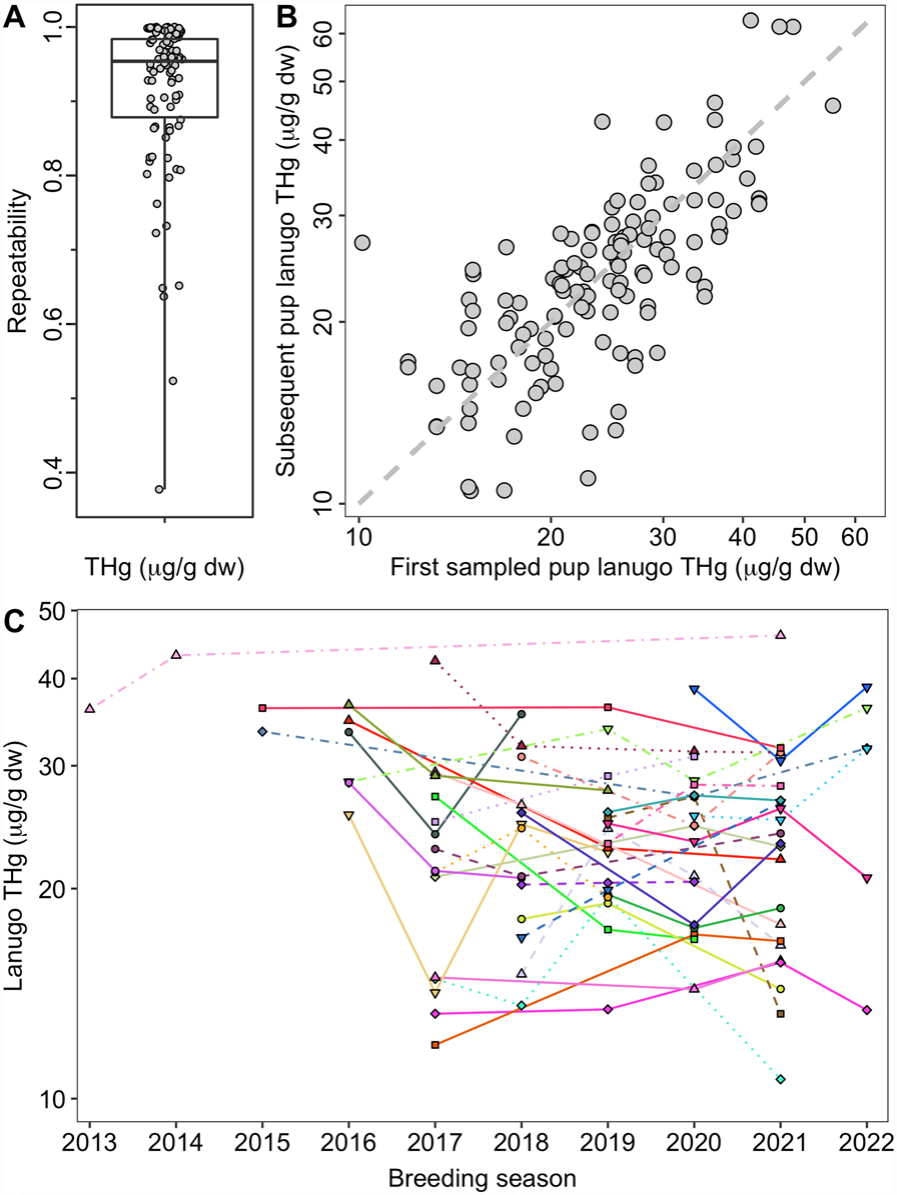
Repeatability of maternal mercury transfer. (**A**) Northern elephant seal (*Mirounga angustirostris*) pup lanugo (hair grown in utero) samples were used to examine the repeatability of total Hg (THg) concentrations in pups of the same female over time (*n* = 90 females). Repeatability values range from 0 to 1, with greater repeatability indicated by values closer to 1. Refer to the Materials and Methods section for calculation of repeatability. In the boxplot, the box delineates the interquartile range, the horizontal line shows the median value, and the whiskers encompass the full range of data, with individual datapoints shown as circles. (**B**) Subsequently sampled elephant seal pups are shown relative to when the first pup sample was obtained for a female. Note that pups were not always sampled in consecutive years, and this figure shows all possible combinations of the first pup sampled and all subsequent pups. (**C**) The subset of adult females between the breeding seasons of 2013 and 2022 are shown that had THg concentrations quantified in at least 3 pups (*n* = 33 females). Successively sampled pups from the same female are shown with the same symbol and color and are connected with the same line type.

Total Hg concentrations in pup lanugo from the same female were moderately repeatable based on the population level repeatability analysis; 72% (95% CI for *R* = 0.61–0.80; *n* = 90 females) of the total variance in pup lanugo THg concentrations was accounted for by differences among and not within individual females (Fig. 4). We observed a range of individually calculated repeatability estimates; however, 92% of individual adult females demonstrated strong repeatability in pup lanugo THg concentrations (Fig. 4). Individually calculated repeatability values were not related to the number of pups sampled per female (2–4 pups per female; Spearman’s *ρ* = − 0.16, *p* = 0.14) nor the maximum number of years between samples (1–8 years; Spearman’s *ρ* = − 0.07, *p* = 0.50).

### Influence of maternal age and year on pup lanugo THg concentrations

**Figure 5:**
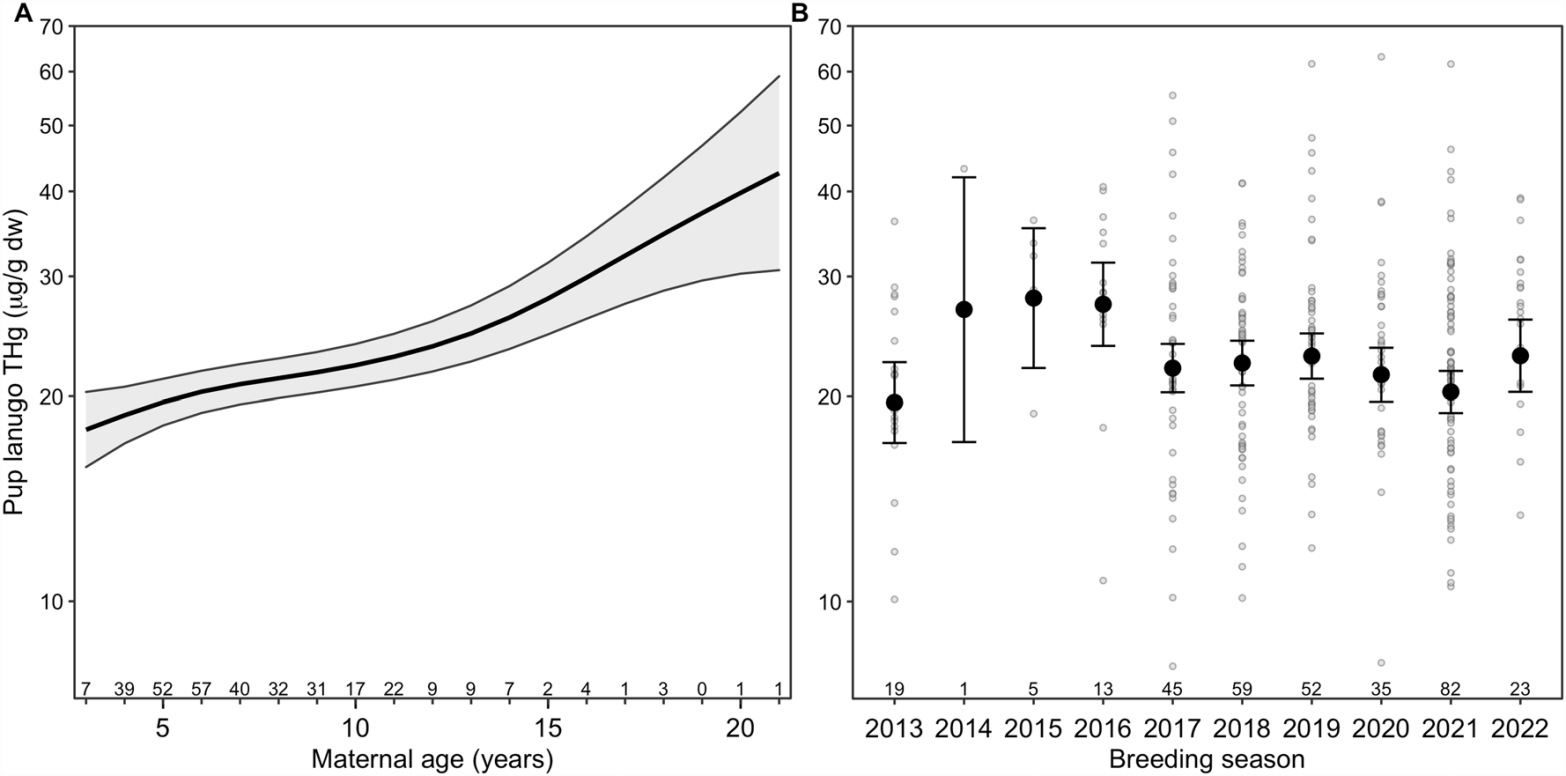
Female age and year affect maternal transfer of mercury. Total Hg (THg) concentrations (μg/g dry weight) in northern elephant seal (*Mirounga angustirostris*) pup lanugo as a function of maternal age and year (*n* = 334 pups from 221 adult females) from a generalized additive mixed model with individual adult female seal as a random effect. Pup lanugo was grown in utero during the approximately 7-month-long maternal foraging trip in the North Pacific Ocean and collected on land during the breeding season between January and March; thus, the samples collected during the breeding season reflect lanugo growth and maternal mercury transfer when females were foraging at sea during the prior year. (**A**) The model-generated predictions for pup lanugo THg concentrations by maternal age are shown for 2021 (26.5% of samples) with 95% confidence limits shaded in gray. Sample sizes by maternal age are shown above the x-axis tick marks. (**B**) Model-generated predictions with 95% confidence intervals are shown for 6-year-old females (18.1% of samples) in each breeding season from 2013 to 2022. Statistical analyses were conducted on log_e_-transformed THg concentrations but the relationships are plotted on log_10_ axes for visualization. Raw data are shown as light gray circles. Sample sizes for each breeding season are shown above the x-axis tick marks.

Elephant seal pup lanugo THg concentrations increased with maternal age (*F* = 10.87, *p* < 0.001, *n* = 334 pups; Fig. 5). Pup lanugo THg concentrations increased by 93.7% between the average 3-year-old and 18-year-old breeding female in the population, based on model predictions for 2021 and accounting for differences among individual females. However, changes in lanugo THg concentrations with maternal age were non-linear over the full range of ages (Fig. 5). For example, we predicted pup lanugo THg concentrations to increase by 13.6% for the first three years of breeding (3 to 6 years). In contrast, pup lanugo THg concentrations were predicted to increase by 6.8% from age 6 to 9 and 24.4% from age 15 to 18.

After accounting for maternal age and individual breeding female, pup lanugo THg concentrations varied among sampling years. The highest mean lanugo THg concentrations were observed during the breeding seasons of 2015 and 2016 (Fig. 5), representing maternal burdens and THg exposure during the foraging trips of 2014 and 2015.

### Pup lanugo THg concentrations related to maternal foraging behavior

#### Foraging behavior cluster analysis

**Figure 6:**
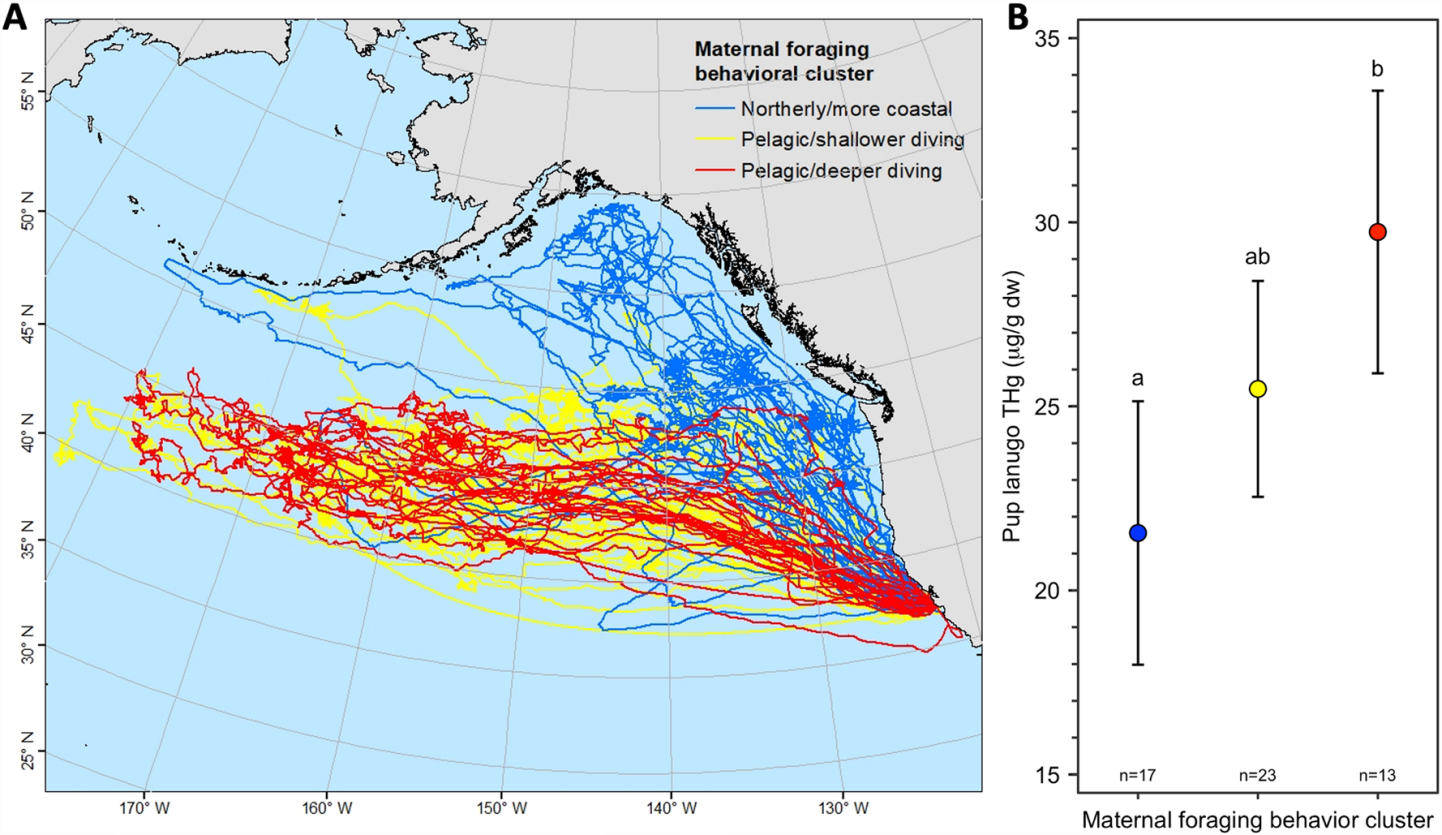
Maternal deep-ocean foraging strategies affect pup mercury concentrations. Total Hg (THg) concentrations in the lanugo (hair grown in utero) of northern elephant seal (*Mirounga angustirostris*) pups (*n* = 53) differed among 3 clusters of maternal foraging behavior that were defined using a hierarchical analysis on principal components. The principal components analysis variables included maternal at-sea locations, diving behavior, and stable isotopes (refer to Table 2 for the complete variable list). (**A**) Individual adult female foraging tracks color-coded by foraging behavior cluster. (**B**) Model-estimated least squares mean pup lanugo THg concentrations with 95% confidence intervals are shown by foraging behavior cluster. Letters above model-estimated least squares mean lanugo THg concentrations indicate significant differences between behavioral clusters.

Pup lanugo THg concentrations differed among the 3 maternal foraging behavior clusters we identified using geographic location variables, diving variables, and stable isotopes (*F*_2,28.2_ = 4.91, *p* = 0.015; Fig. 6). Generally, pups of females that dove deepest during the day and night (both median and 90th quantile of dive depths) and traveled farthest west across the Pacific Ocean (pelagic and deeper diving cluster) had 37.5% higher lanugo THg (model-estimated least squares mean) concentrations than the pups of females that stayed closer to the continental shelf, dove to shallower foraging depths during the day (median and 90th quantile of dive depths), remained farthest east, and traveled farther north throughout their foraging at sea (northerly and more coastal cluster; *t* = 3.15, *p* = 0.003; Table 2). Pups from the middle cluster, with lanugo THg concentrations that overlapped the other two clusters (*t* ≥ 1.70, *p* ≤ 0.10), were the offspring of females that were intermediate to the other two behavioral clusters for many of their locations and diving behavioral metrics (pelagic and shallower diving cluster).

#### Maternal predictor variables for individual pup lanugo THg concentrations

At the individual level, pup lanugo THg concentrations increased with both maternal *δ*^13^C values and the median depth of day foraging dives, after accounting for differences among years (Table 3). Two additional models, identical to the top model except for adding maximum latitude or minimum longitude, were considered for biological importance (ΔAIC_c_ ≤ 2.0 of the top model). However, both variables were determined to be uninformative parameters^80^. These top three models comprised a cumulative 45% of total model weights. We did not observe support for any additional variables (Table 3) in explaining the variability of individual pup lanugo THg concentrations.

**Table 3:** Multi-model inference table.

Model	k	− 2LogL	AIC_c_	ΔAIC_c_	*w*_i_	Evidence ratio
**Year + δ**^**13**^**C + day dive** **depth**	**12**	**302.79**	**334.59**	**0.00**	**0.25**	**1.00**
Year + δ^13^C + day dive depth + longitude	13	301.06	336.40	1.80	0.10	2.46
Year + δ^13^C + day dive depth + latitude	13	301.10	336.44	1.84	0.10	2.51
Year + δ^13^C + day dive depth + age	13	301.55	336.88	2.29	0.08	3.14
Year + δ^13^C + day dive depth + night dive depth (90th quantile)	13	301.73	337.06	2.47	0.07	3.43
Year + δ^13^C + day dive depth + night dive depth	13	302.20	337.53	2.94	0.06	4.34
**δ**^**13**^**C + day dive depth**	**4**	**332.62**	**341.45**	**6.86**	**0.01**	**30.83**
**Year + day dive depth**	**11**	**316.37**	**344.81**	**10.22**	**0.00**	**165.27**
**Year + δ**^**13**^**C**	**11**	**327.18**	**355.62**	**21.03**	**0.00**	**3.68 × 10**^**4**^
Null model	2	361.76	366.00	31.41	< 0.01	6.60 × 10^6^

Model selection results to explain the variability in northern elephant seal (*Mirounga angustirostris*) pup lanugo THg concentrations sampled at the Año Nuevo colony (2013 to 2021 breeding seasons; California, USA) based on a set of at-sea maternal foraging behavior variables. Year was a categorical variable representing the 9 different years of satellite-tracked foraging trips, *δ*^13^C (‰) was quantified in maternal red blood cells, latitude was the northernmost latitude (º) reached during the foraging trip, longitude was the westernmost longitude (º) reached during the trip, and age was maternal age when the pup sample was collected. Median day and night foraging dive depths and the 90th quantile of night foraging dive depths were also included as behavioral variables. Models in the table represent all models with a ΔAIC_c_ < 4 as well as the null model and all models with just one variable removed from the top model (lowest AIC_c_ value). Each model has the following terms reported: k (number of parameters in the model), − 2LogL (− 2 × log(likelihood)), AIC_c_ (Akaike information criterion adjusted for small sample sizes), ΔAIC_c_ (difference in the AIC_c_ value between the top model and the model of interest), *w*_i_ (Akaike model weight), evidence ratio (*w*_i_ of top model/*w*_i_ of the model of interest; likelihood of the top model over the model of interest).

Bolded models are the top model and those that are the same as the top model, but have a single variable removed.

**Figure 7:**
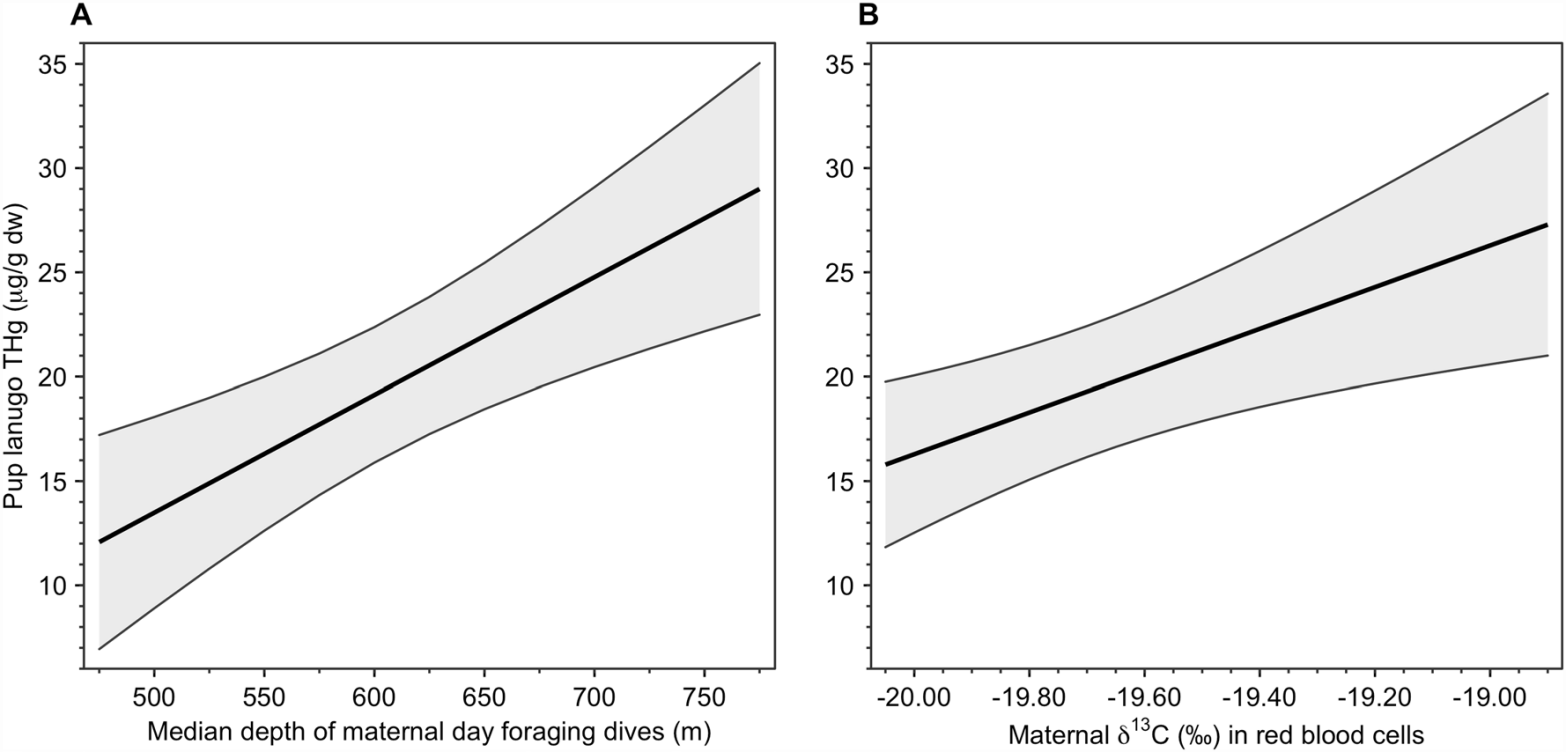
Pup mercury concentrations correspond to maternal foraging behaviors. Total Hg (THg) concentrations in the lanugo (hair grown in utero) of northern elephant seal (*Mirounga angustirostris*) pups (*n* = 53) increased with increasing (**A**) median depth of maternal foraging dives during the day and (**B**) maternal *δ*^13^C values in red blood cells. Adult female northern elephant seals forage continuously at sea during the entire gestation period, only returning to land at the end of an approximately 7-month-long foraging trip to give birth, when we sampled maternal blood and pup lanugo for THg concentrations and stable isotopes. Model-averaged predictions with 95% confidence intervals were generated from the complete set of candidate models for 6-year-old females from the 2013 breeding season, with all non-focal covariates (refer to Table 3 for the full covariate list) held at their median values.

Pup lanugo THg concentrations increased by 140.2% over the approximate range of observed median maternal day dive depths (475 to 775 m), with an increase of 2.8 μg/g dw for each 50 m increase in median day diving depth, based on model-averaged predictions using the median maternal *δ*^13^C value of − 19.53‰ and median values for all other variables (Fig. 7). When day diving depth was held at the median value (633 m), the mean pup lanugo THg concentration increased 72.8% over the approximate range of maternal *δ*^13^C values (− 20.05 to − 18.90‰), with an increase of 1.0 μg/g dw for each 0.1‰ increase in *δ*^13^C values (Fig. 7).

## Discussion

Highly mobile and deep-diving adult female northern elephant seals consume prey contaminated with mercury from mesopelagic food webs and predictably transfer bioaccumulated mercury into their pups during gestation. Notably, although pup lanugo and pup blood mercury concentrations increased with maternal mercury contamination, the slopes of these relationships (< 1) indicated that the proportion of maternal mercury that was transferred to pups during gestation decreased with increasing maternal blood mercury concentrations, similar to observations in birds and frogs^6,7,9^. Gestational transfer of mercury occurs at a vulnerable life history stage and is the starting point for pup mercury bioaccumulation; thus, quantification of factors that influence the maternal transfer of mercury is essential to better understanding ecotoxicological risk. We found that mercury bioaccumulation in the lanugo of developing seal pups was related to maternal mercury bioaccumulation, increased with maternal age, and was strongly associated with maternal foraging behavior.

The present study demonstrated that mercury concentrations in maternal blood (early lactation) and pup lanugo can be used to represent mercury exposure and maternal transfer of mercury to pups during the extensive foraging trips of northern elephant seals. Importantly, pup lanugo mercury concentrations reflect at-sea maternal mercury levels because lanugo is grown in utero while females are on their foraging trips. Mercury concentrations in lanugo are not influenced by the physiological mechanisms accompanying fasting and lactation after parturition, unlike pup or maternal blood. We expect the most robust relationship between pup lanugo and maternal blood to occur at birth because lanugo (grown in utero) mercury concentrations would not change after birth, whereas the whole blood mercury concentrations of adult females increased by 103% over 18 days during lactation (between early and late lactation samples) in response to significant declines in body mass during fasting^26^. However, maternal blood mercury still explained approximately 60% of the variation in lanugo mercury concentrations when mother–pup pairs were sampled 20% of the way through lactation (after females had fasted on land for 5 to 15 days upon returning from their foraging trip).

Although pup blood mercury concentrations declined by 54% as pups rapidly increased in body mass from 5 to 23 days post-parturition, pup blood mercury burdens simultaneously increased by 25% over this 18-day lactation period (range: 0.15–0.35 mg mercury). This increase in the mercury blood burden represents a substantial transfer of mercury via lactation to nursing pups, despite the lack of a correlation between milk mercury concentrations and maternal blood mercury concentrations observed in grey seals (*Halichoerus grypus*) and northern elephant seals^12,13^. Pups are estimated to consume 137.7 kg of milk during lactation^82^. Therefore, even relatively low concentrations of mercury in milk (29.5 ng/g ww, averaged between early and late lactation milk samples^12^) can result in an estimated total maternal transfer of 4.06 mg of mercury via milk to pups during lactation. During this same lactation period, adult female blood mercury concentrations rapidly increase even though there is no external source of mercury through diet because they are fasting and lose 30% of body mass (including both lipid and protein stores)^26,27^. These results underscore the importance of calculating both the quantity of mercury as well as tissue mercury concentrations, such as in blood, that typically decrease while pups are nursing and undergoing a period of rapid growth^12–14^. The mercury burden of pups represents an early starting point of contamination that could increase the likelihood of future physiological, reproductive, or survival impacts. 

Surprisingly, the firstborn pup did not receive a higher dose of mercury than the subsequent pup from the same female in a future year, a question that, to our knowledge, has not previously been investigated in free-ranging mammals. Further, the pups of different individual females were far more variable in mercury exposure than among pups from the same female. In capital-breeding cetaceans, a strategy shared with elephant seals, the first offspring generally receives a heightened transfer of a persistent organic pollutant contaminant burden because the female has accumulated persistent organic pollutants in blubber up until that point in time with limited offloading pathways^22^. Subsequent offspring have decreased maternal transfer of persistent organic pollutants^22^. The absence of an increase in the maternal transfer of mercury to the firstborn pups of northern elephant seal females may partly be due to different toxicokinetics of mercury in comparison to persistent organic pollutants and the life history characteristics of northern elephant seals. The lipophilic nature of persistent organic pollutants compared with the protein-bound nature of mercury means that these contaminants tend to be stored in different tissues. Thus, mercury and persistent organic pollutants can differ in bioavailability and sequestration pathways when different tissues undergo catabolism or growth. For example, northern elephant seals can offload substantial amounts of mercury annually into hair during the molt. Furthermore, adult females begin reproducing at a relatively young age and often do not reach full body size until after they have undergone multiple breeding seasons^60,61^; the continuation of adult growth during reproduction may influence where mercury consumed from prey is stored within the body and how much mercury is available for maternal transfer. The mean annual natality rate is estimated between 84 and 87%^45,83^, suggesting that most females have a pup every year, and the oldest females are still breeding past the age of 20^61^.

Mercury concentrations in northern elephant seal pup lanugo increased non-linearly with maternal age, suggesting that the pups of older females will be exposed to higher mercury concentrations during development. Few studies have quantified mercury levels in known-age female vertebrates or their offspring across a full range of maternal ages. Therefore, the relationship between adult female mercury contamination and age is poorly described and is equivocal. Mercury concentrations increased with age in the livers of stranded known-age female Saimaa ringed seals (*Pusa hispida saimensis*)^84^, the muscle, livers, and kidneys of stranded adult northern fur seals (*Callorhinus ursinus*)^85^, in whole blood of bottlenose dolphins (*Tursiops truncatus*)^86^, and the livers and hair of Eurasian otters (*Lutra lutra*)^87^, but did not increase with age in mature adult striped dolphins (*Stenella coeruleoalba*)^88^ or wandering albatross (*Diomedea exulans*)^89,90^. Although the northern fur seal data^85^ were analyzed linearly and did not separate males and females, the data appear to have a non-linear shape with a pronounced increase in the oldest individuals, similar to that observed for northern elephant seals.

At-sea maternal behavior is likely the most critical driver of maternal transfer of mercury to northern elephant seal pups during gestation. This is demonstrated by clear links between pup mercury and maternal foraging behavior (Figs. 6–7) and the high repeatability in pup mercury of individual females. After accounting for maternal age, we identified that higher pup lanugo mercury concentrations were associated with deeper diving females. Additionally, pup lanugo mercury concentrations were higher in females that foraged on prey enriched in^13^C, after accounting for foraging depth and geographic location. Mesopelagic prey can become enriched in^13^C as a result of oceanographic and biological processes related to the depth and origin of the food web as well as increasing trophic level^91^. Similar links between mercury exposure to offspring and maternal foraging behavior have been found in Steller sea lions (*Eumetopias jubatus*)^50^. Few northern elephant seal females survive to produce more than one or two pups, and those that survive longer contribute the most pups to the population; specifically, females that produced 10 pups over their life comprised 6% of all females but produced 55% of all pups at the Año Nuevo colony^61^. It is possible that increased mercury bioaccumulation may result from foraging strategies that confer higher adult female survival rates, which could in part explain why mercury concentrations were higher in the pups of older females. Importantly, the mechanisms behind these relationships remain unclear because there has been only limited analysis examining links between lifetime female reproductive success, female survival, and foraging behavior of northern elephant seals. Because a small subset of highly successful female elephant seals within the northern elephant seal population contributes substantially more pups than the remainder of the breeding females (described as supermoms^61^), the foraging behavior and mercury contamination of the most successful females may have disproportionate consequences for the risk of adverse mercury effects in elephant seal pups. Although toxicological benchmarks have not been well validated for pinnipeds, 20 μg/g dw and 30 μg/g dw in hair have been proposed for mammals as a level of concern for sublethal health effects^50,92^, and as a threshold for clinical health effects^92^, respectively. Raw data from this study indicate that 93% of pups sampled from females > 12 years old exceeded the benchmark of 20 μg/g dw, compared with 67% of sampled pups born to females ≤ 12 years old. Similarly, 64% of pups born to females > 12 years old exceeded the benchmark of 30 μg/g dw, compared with 14% of pups from females ≤ 12 years old.

**Figure 8:**
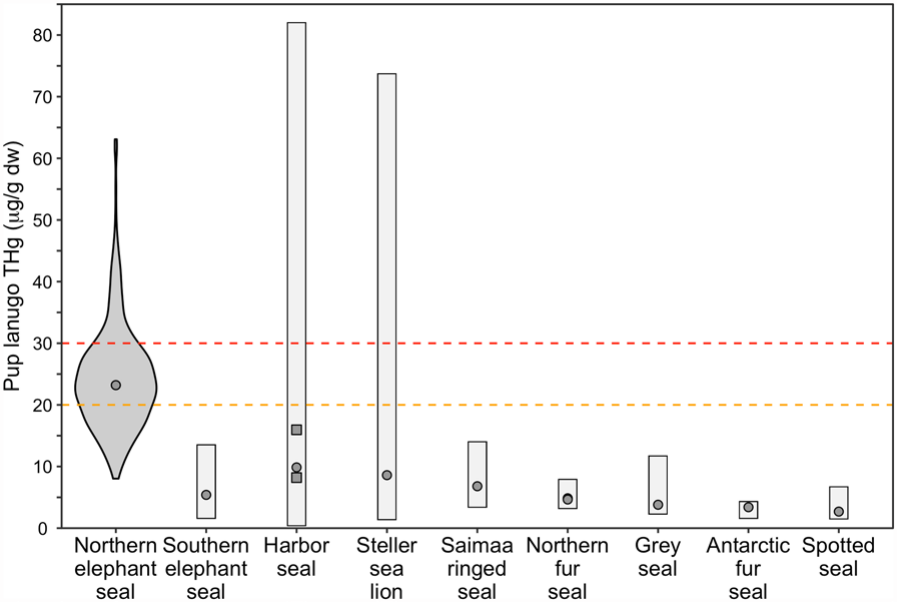
Hair mercury concentrations in seal and sea lion pups. Total Hg (THg) concentrations (µg/g dw) in the lanugo (hair grown in utero) of northern elephant seal (*Mirounga angustirostris*) pups from the present study and representative studies quantifying lanugo THg concentrations in seal and sea lion pups worldwide^13,51,84,95,106–110^. For northern elephant seal pups from the present study, a violin plot shows the distribution of THg concentrations. Circles represent median THg concentrations for all individual studies (except for two harbor seal, *Phoca vitulina*, studies where only arithmetic mean THg concentrations were presented; delineated by gray squares) and the range of THg concentrations observed for each species is delineated by the gray bars. The dashed orange line at 20 µg/g dw indicates the concentration that has been proposed for mammals as a level of concern for sublethal health effects in mammals^50,92^, and the dashed red line at 30 µg/g dw indicates the proposed threshold for clinical health effects in mammals^92^. Note that for the harbor seals, lanugo and non-lanugo hair was sampled from pups because they are relatively unique among pinnipeds and typically molt their lanugo in utero before birth.

Young and developing animals are particularly vulnerable to contaminant exposure, especially mercury^93,94^. Our results demonstrated relatively high mercury concentrations in lanugo and blood of northern elephant seal pups compared with representative studies of other seal and sea lion pups worldwide (Fig. 8). This suggests a substantial exposure in utero from maternal transfer while adult females are foraging in the mesopelagic North Pacific, although individual pups demonstrated high variability in both lanugo mercury concentrations (8.03–63.09 μg/g dw) and blood mercury concentrations (0.09–0.35 μg/g ww). In the present study, 70% of northern elephant seal pups exceeded 20 μg/g and 18% exceeded 30 μg/g. In comparison, 25% of Steller sea lion pups from the western Aleutian Islands exceeded the toxicological benchmark of 20 μg/g, and this population of Steller sea lions is considered to be at risk from adverse effects of mercury^95^. A female with blood THg concentrations higher than 0.28 μg/g ww was likely to have a pup with at least 20 μg/g dw in lanugo, and a female with blood higher than 0.46 μg/g ww was likely to have a pup with at least 30 μg/g dw in lanugo. Based on these thresholds and other marine mammal studies that observed non-lethal effects at relatively low tissue THg concentrations^35,96^, northern elephant seal pups may be at a high risk of non-lethal adverse health effects during early development.

The maternal transfer of mercury to pups also represents a potential bio-transport mechanism to move mercury from the mesopelagic ocean to nearshore and terrestrial ecosystems, in addition to the transport across these systems by adult seals^97^. A portion of the mercury transferred to pups may be excreted into the ecosystem via feces^98,99^, but much of the mercury in blood will likely be integrated into newly formed tissue as the nursing seals rapidly gain mass. Elephant seal pups may therefore represent a significant step in the bio-transport pathway. The average mortality of northern elephant seal pups, calculated from 1984 to 2020 at the mainland Año Nuevo colony, was 7.2%; thus, pup mortality based on the average number of pups born annually from 2010 to 2020 would result in the annual delivery of mercury into nearshore and terrestrial ecosystems^97^ from 113 carcasses as well as from the lanugo of 1575 pups^100^. In 2010, approximately 40,684 northern elephant seal pups were born at colonies in California. With a mortality estimate of 7.2%, 2900 pups would annually contribute their mercury burdens to the ecosystems around northern elephant seal breeding colonies in California.

The geographic locations and depths in the mesopelagic North Pacific from which northern elephant seals obtain higher mercury contamination from their prey will pose an increased risk to cryptic mesopelagic-associated species that forage at similar or higher trophic levels, including cetaceans, sharks and tuna^101,102^. Furthermore, after accounting for maternal age, the inter-annual variability in pup lanugo mercury concentrations suggests that mercury availability within mesopelagic North Pacific food webs may vary among years, which could result in greater mercury exposure during gestation for some cohorts of elephant seal pups. Inter-annual variability in mesopelagic mercury also has implications for mercury exposure and toxicological risk at an ecosystem scale. During this study, an extended marine heatwave (2014 to 2017) resulted in anomalously warm subsurface water that extended down to the bottom of the mesopelagic zone (1000 m), with peak subsurface temperatures and deeper diving seal behavior observed during the foraging trip that preceded the 2016 breeding season^103^. Deeper diving behavior could alter mercury bioaccumulation through differences in diet with depth. The 2016 breeding season coincided with one of the highest annual mean pup lanugo mercury concentrations we observed. It is currently unclear how methylation rates or the bioavailability of mercury in mesopelagic food webs may be related to changes in oceanographic conditions such as temperature and salinity, although warming oceans are theorized to change methylmercury cycling within marine food webs^104,105^. Thus, further exploration of the mechanisms that dictate methylmercury availability in mesopelagic food webs may provide better linkages between deep-sea mercury contamination and toxicological risk for mesopelagic predators and their offspring.

## Data Availability

The datasets analyzed during the current study^81^ are available in the ScienceBase repository, https://doi.org/10.5066/P14UANSS.
